# Exogenous recombinant human growth hormone effects during suboptimal energy and zinc intake

**DOI:** 10.1186/1743-7075-2-10

**Published:** 2005-04-07

**Authors:** Russell Rising, Julio F Scaglia, Conrad Cole, Rozalia Tverskaya, Debora Duro, Fima Lifshitz

**Affiliations:** 1 EMTAC Inc., 514 Santander Ave, #5, Coral Gables, FL 33134 USA; 2 Lawndale Medical Clinic, 7109-B Lawndale Ave., Houston, TX 77023 USA; 3 Department of Pediatrics, Emory University, 2040 Ridgewood Drive NE, Atlanta, GA 30322 USA; 4 71-50 Parsons Blvd, #5B, Flushing, NY 11365 USA; 5 Children's Hospital Boston, Dept. of Gastroenterology, 300 Longwood Avenue, Boston, MA 02115 USA; 6 Sansum Medical Research Institute, 2219 Bath Street, Santa Barbara, CA 93105 USA

## Abstract

**Background:**

Energy and Zinc (Zn) deficiencies have been associated with nutritional related growth retardation as well as growth hormone (GH) resistance. In this study, the relationship between suboptimal energy and/or Zn intake and growth in rats and their response to immunoreactive exogenous recombinant human GH (GHi), was determined.

**Results:**

Rats treated with GHi and fed ad-libitum energy and Zn (100/100) had increased IGFBP-3 (p < 0.05) as compared with NSS (215 ± 23 vs. 185 ± 17 ng/ml) along with similar body weight gain. Rats treated with GHi and fed suboptimal energy and full Zn (70/100) had significantly increased weight gain (109.0 ± 18.2 vs. 73.8 ± 11.0 g) and serum IGF-I levels (568 ± 90 vs. 420 ± 85 ng/ml), along with decreased total body water (TBW; 61.0 ± 1.6 vs. 65.7 ± 2.1%) as compared to NSS controls. However, body weight gain was reduced (p < 0.05) as compared with rats fed ad-libitum energy. Growth hormone treated rats fed only suboptimal Zn (100/70), had increased weight gain (217.5 ± 13.2 vs. 191.6 ± 17.9 g; p < 0.05) compared to those given NSS. These rats gained weight in similar amounts to those fed full Zn. Rats treated with GHi and fed both suboptimal energy and Zn (70/70) showed similar results to those fed suboptimal energy with appropriate Zn (70/100), along with significant increases in IGFBP-3 levels (322 ± 28 vs. 93 ± 28 ng/ml). All restricted rats had reduced 24-h EE (kcal/100 g BW) and physical activity index (oscillations/min/kg BW) and GHi did not overcome these effects.

**Conclusion:**

These results suggest that GHi enhances weight gain in rats with suboptimal energy and Zn intake but does not modify energy expenditure or physical activity index. Suboptimal Zn intake did not exacerbate the reduced growth or decrease in energy expenditure observed with energy restriction.

## Introduction

Nutritional growth retardation or nutritional dwarfing (ND) denotes a linear growth deterioration accompanied by inadequate weight gain [1]. It has been associated with short stature since growth deceleration represents an adaptive response to suboptimal nutrition. In the United States, ND is frequently associated with self imposed dieting due to fear of obesity and/or hypercholesterolemia, extreme exercise and eating disorders, among many other reasons [2-4]. In contrast to more severe forms of malnutrition observed worldwide, ND is manifested by a height-for-age deficit while both weight-for-height and standard biochemical nutritional indexes remain within normal limits [1]. Several reports have also demonstrated reduced Insulin-like Growth Factor-1 (IGF-I) level and decreased erythrocyte Na^+^K^+^-ATPase activity during suboptimal energy intake [5-9]. The degree of energy restriction portrays one of the most important factors related with growth hormone (GH) action. Consequently, elevated GH level along with reduced IGF-I, Insulin-like Growth Factor Binding Protein-3 (IGFBP-3) and Insulin levels have been described in both human and animal research as a consequence of starvation [10-18]. Furthermore, attenuated spontaneous GH secretion has also been reported in patients with ND during puberty [19]. However, utilizing an animal model of suboptimal nutrition, it has been reported that GH effects are feasible during mild energy restriction by administering exogenous GHi [20,21].

Although adequate energy intake plays a major role affecting growth, micronutrients like Zn have also demonstrated to be essential regulators of growth and GH action [22-24]. Profound Zn deficiency with adequate energy intake has been frequently associated with growth failure and delayed sexual maturation, affecting cell division, DNA, RNA and protein synthesis [25,26]. Zinc deficiency also reduces the level of liver GH receptors, serum IGF-I, GH binding proteins and both mRNA's for GH receptors and IGF-I [24,26]. Furthermore, binding of Zn in cultured rat hepatocytes with a Zn chelator DTPA (diethylenetriaminepenta-acetic acid) did not reduce mRNAs for IGF-I, GH receptors or GH binding proteins while metallothionein gene expression was strongly inhibited. Therefore, the decline in IGF-I associated with in vivo Zn deficiency did not appear to be due to changes in extracellular Zn concentration at the hepatocyte level [27]. Often Zn deficiency is associated with reduced energy intake [28,29] and the effects of each one of these nutritional alterations alone, or in combination with each other is not known. Furthermore, the effects of GHi administration in rats with experimentally induced suboptimal energy with and without decreased Zn intakes have not been elucidated.

In this paper we report the effects of exogenous GHi administration in an animal model of suboptimal nutrition where energy and/or Zn intake was decreased by 30% of requirements [5,20,21]. Our goal was to determine if exogenous GHi administration would attenuate the detrimental effects of mild restrictions of energy and Zn individually or in combination with each other. Furthermore, we hypothesized that energy expenditure and physical activity would be reduced with mild nutrient restrictions. This was determined utilizing a new Enhanced Metabolic Testing Activity Chamber.

## Results

### Effects of GHi on weight gain

**Figure 3:**
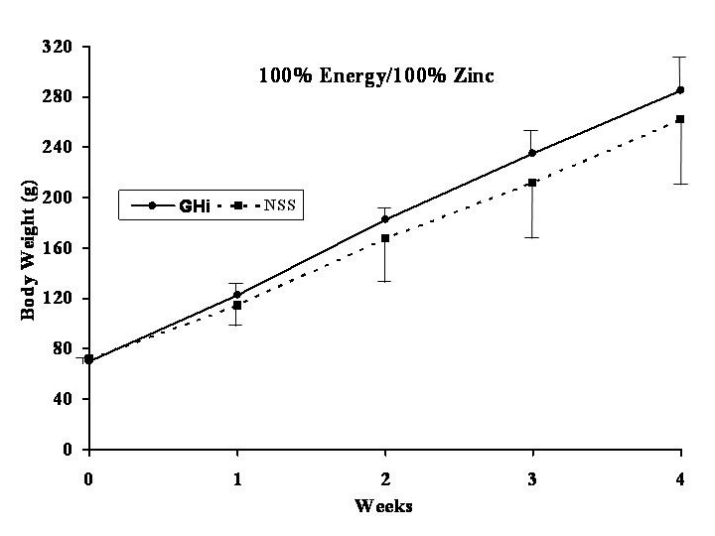
Weekly weight gain for both growth hormone treated (filled dots, continued line) and normal saline control rats (filled squares, interrupted line) fed a 1:1 carbohydrate to fat purified diet with 100% of the energy and 100% of the zinc requirement for rats. * = significant difference (p < 0.05) for that particular week.

**Figure 4:**
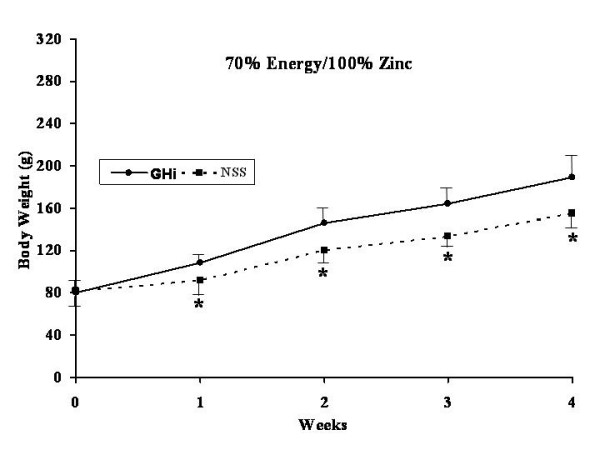
Weekly weight gain for both growth hormone treated (filled dots, continued line) and normal saline control rats (filled squares, interrupted line) fed a 1:1 carbohydrate to fat purified diet with 70% of the energy and 100% of the zinc requirement for rats. * = significant difference (p < 0.05) for that particular week.

**Figure 5:**
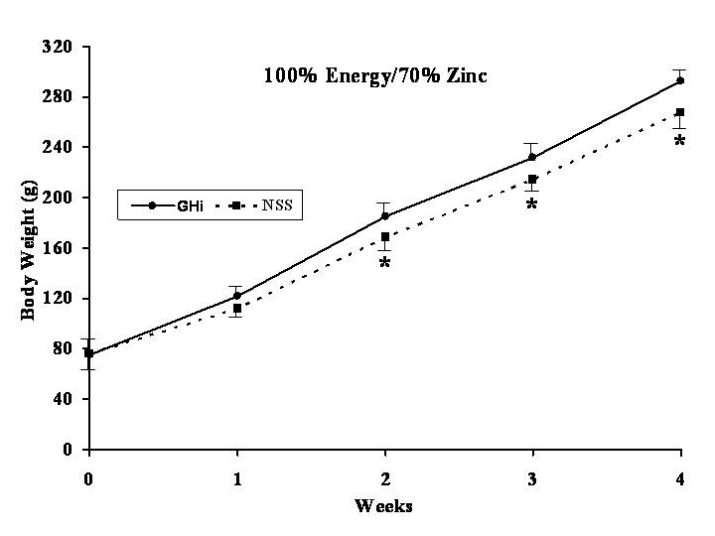
Weekly weight gain for both growth hormone treated (filled dots, continued line) and normal saline control rats (filled squares, interrupted line) fed a 1:1 carbohydrate to fat purified diet with 100% of the energy and 70% of the zinc requirement for rats. * = significant difference (p < 0.05) for that particular week.

**Figure 6:**
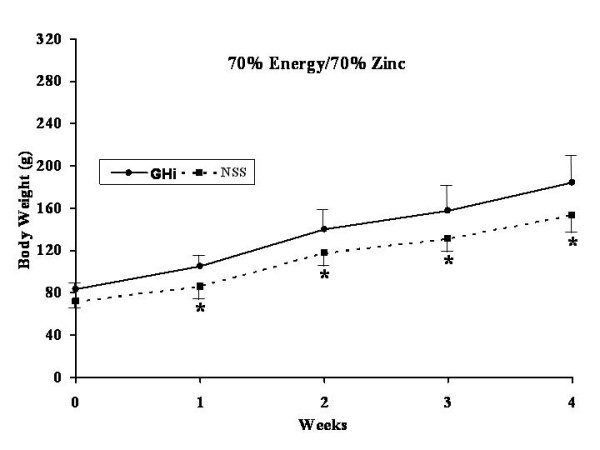
Weekly weight gain for both growth hormone treated (filled dots, continued line) and normal saline control rats (filled squares, interrupted line) fed a 1:1 carbohydrate to fat purified diet with 70% of the energy and 70% of the zinc requirement for rats. * = significant difference (p < 0.05) for that particular week.

All rats were healthy throughout the experiment without any apparent illness or weight loss. The effects of GHi administration (solid lines) versus NSS administration (dotted lines) for each dietary group are shown in Figures 3, 4, 5, 6.

No significant differences were found in body weight gain between the GHi treated and the NSS control group rats fed 100% energy/100% Zn (Diet A) (Figure 3). However, rats fed 70% energy/100% Zn (Diet B) and treated with GHi showed significantly (p < 0.05) increased weight gain over the four week experimental period as compared with their respective NSS controls. The differences in body weight gain became apparent after the first week of the study (Figure 4). However, the rats in this group fed restricted energy and full Zn gained less weight than the rats fed energy and Zn ad-libitum (Diet A) regardless of GHi treatment. Similarly growth hormone administration produced a response in the group fed 100% energy/ 70% Zn (Diet C) were GHi treated rats gained significantly (p < 0.05) more weight than their respective NSS controls (Figure 5). The differences became apparent after the second week of the study. However, the overall weight gain of the rats in this group fed restricted amounts of Zn and a full energy complement was similar to those in the group fed energy and Zn ad-libitum (100% energy/100% Zn; Diet A) regardless of GHi treatment. Finally, when both energy and Zn intake were reduced to 70% energy/70% Zn (Diet D), rats treated with GHi gained significantly (p < 0.05) more weight than their respective NSS controls (Figure 6). Weight gain differences became apparent after the first week of the study. The weight gain of the rats in this group was similar to that found for rats restricted to 70% of the ad-libitum energy intake with full Zn intake (Diet B).

### Effects of GHi on IGF-I, IGFBP-3 and insulin levels

The hormonal pattern at the end of the study is shown in Table 3. When rats were fed 100% energy/100% Zn (Diet A) and treated with GHi, IGFBP-3 increased significantly (p < 0.05) versus those rats given NSS. In comparison to the NSS treated rats, treatment with GHi increased serum IGF-I in both groups of rats fed 70% ad-libitum energy with or without Zn restriction (Diets B and D). Furthermore, serum IGFBP-3 increased in the GHi-treated group when both energy and Zn intake were reduced to 70%. However, serum IGFBP-3 did not differ among GHi-treated rats fed at 70% of energy intake and 100% of Zn. Additionally, no significant differences were found in serum IGFBP-3 when energy was maintained at 100%. Serum insulin did not change significantly in GHi treated rats as compared to controls.

**Table 3:** Hormonal profile after mild energy and Zn restriction in rhGH treated rats

Energy/zinc (%)	100/100		70/100		100/70		70/70	
	rhGH^1^	NSS^2^	rhGH	NSS	rhGH	NSS	rhGH	NSS

IGF-1 (ng/ml)	1012.5 ± 82.7	784.8 ± 231.3	568.4 ± 90.2*	420.3 ± 84.5	1089.5 ± 133.9	1074.1 ± 220.1	657.0 ± 114.1*	423.0 ± 139.9
IGFBP-3 (ng/ml)	214.5 ± 23.3*	184.7 ± 17.2	107.9 ± 35.5	96.9 ± 9.3	231.0 ± 36.0	201.0 ± 21.3	322.0 ± 28.4*	93.4 ± 28.0
Insulin (mIU/ml)	21.3 ± 7.6	52.7 ± 28.6	22.2 ± 12.6	39.5 ± 20.8	50.9 ± 14.4	71.4 ± 17.3	26.3 ± 6.6	17.1 ± 6.5

* = P < 0.05 between treatment groups

^1 ^= Recombinant human growth hormone

^2 ^= Normal saline solution

### Metabolic changes associated with caloric restriction

In comparison to rats administered NSS, GHi administration had no effects on 24-hour energy expenditure and the index of physical activity within any of the dietary treatments (Table 4). However, the RQ was lower in GHi treated rats fed 70% of ad-libitum energy intake. Energy expenditure (p < 0.05) and physical activity index (p < 0.05) were reduced in all restricted groups as compared with all those fed a full complement of energy. The reduction in energy expenditure was from 19.5 ± 2.9 to 16.9 ± 1.9 kcal/100 g body weight while the reduction in the physical activity index was from 2.5 ± 0.5 to 2.1 ± 0.3 oscillations/min/kg body weight. Mild Zn restriction did not further reduce these parameters below those found for the energy restricted groups. Similarly, GHi administration did not overcome the reduced energy expenditure and physical activity index found in the energy restricted rats.

**Table 4:** Metabolic profile of rats after mild energy and zinc restriction

Energy/zinc (%)	100/100		70/100		100/70		70/70	
Dietary treatment	rhGH^1^	NSS^2^	rhGH	NSS	rhGH	NSS	rhGH	NSS

24-EE^2 ^(Kcal/100 g BW)	21.2 ± 2.4	19.6 ± 3.3	17.2 ± 1.5	16.4 ± 0.7	17.4 ± 3.4	19.8 ± 2.0	17.5 ± 2.8	16.5 ± 2.4
RQ^3 ^(VCO_2_/VO_2_)	0.82 ± .02	0.89 ± .03	0.82 ± .08	0.89 ± .07	0.90 ± .09	0.81 ± .01	0.82 ± 0.1	0.91 ± 0.01
Physical activity^4^	2.6 ± 0.5	2.2 ± 0.2	2.0 ± 0.5	2.2 ± 0.1	2.3 ± 0.4	2.7 ± 0.8	2.3 ± 0.2	2.1 ± 0.3

^1 ^= Recombinant human growth hormone

^2 ^= Normal saline solution

^3 ^= Twenty-four hour energy expenditure

^4 ^= Respiratory quotient

^5 ^= Oscillations/minute/kg body weight

### Effects of GHi on body composition

Body composition analysis is shown in Table 5. There were no significant differences in FFM and BF between the GHi and NSS groups regardless of dietary treatment. However, compared to their respective NSS controls, rats treated with GHi and fed either energy restricted diet, with our without Zn deficiency (Diets B and D), showed significant (p < 0.05) reductions in TBW.

**Table 5:** Body composition by carcass analysis after mild energy and zinc restriction.

Energy/zinc (%)	100/100		70/100		100/70		70/70	
Dietary treatment	GHi^1^	NSS^2^	GHi	NSS	GHi	NSS	GHi	NSS

Body fat (%)	12.0 ± 1.3	12.8 ± 2.1	8.4 ± 2.6	8.3 ± 1.0	12.3 ± 1.4	12.6 ± 2.1	7.2 ± 1.0	8.8 ± 3.0
Fat-free mass (%)	88.0 ± 1.3	87.2 ± 2.2	91.6 ± 2.6	91.7 ± 1.0	88.1 ± 1.4	87.6 ± 0.9	92.8 ± 1.0	91.2 ± 3.0
Total Body water (%)	57.7 ± 3.3	60.3 ± 2.2	61.1 ± 1.6*	65.8 ± 2.1	57.3 ± 2.9	59.9 ± 1.6	58.8 ± 7.2*	66.6 ± 2.1

* = P < 0.05 between treatment groups

^1 ^Immunoreactive recombinant human growth hormone

^2 ^Normal saline solution

## Discussion

The experimental animal model of suboptimal nutrition allowed the independent assessment of suboptimal intake of specific nutrients and the effects of administered exogenous GHi. At the same time we were able to determine the effects of administered GHi under these conditions. In this study different conditions of suboptimal nutrition in rats were assessed by limiting dietary energy and/or zinc intakes to 70% of ad-libitum. This was possible by utilizing purified diets which allowed us to provide a precise amount of all nutrients, including Zn, which might not have been possible with commercial rat chows. Furthermore, this also permits determination of the effects of rhGH administration on 24-hour energy expenditure and on the index of physical activity utilizing an established rodent model of suboptimal nutrition, which has never been studied before. The data showed that exogenous recombinant human growth hormone enhanced body weight gain and increased serum IGF-I levels in energy restricted rats. Furthermore, serum IGFBP-3 levels were also increased during simultaneous suboptimal energy and zinc restriction. However, suboptimal zinc restriction alone did not interfere with body weight gain or exacerbate any of the detrimental effects of specific suboptimal energy restriction. Moreover, GHi treatment reduced the amount of total body water in energy restricted rats. Finally, a reduction in energy expenditure and the index of physical activity were shown during suboptimal energy intake and Zn restriction however, GHi treatment did not enhance these parameters. The rat's physical activity was not restricted in any way. The slightly smaller space provided by the Nalgene metabolic cage might have potentially reduced the rat's spontaneous physical activity, however this appeared not to affect the results. All rats had metabolic measurements under the same conditions.

Decreased body gain was previously reported during mild suboptimal energy intake in rats fed at 60 and 80% of ad-libitum energy intake [5]. These findings showed that a 40% reduction in energy intake reduced weight gain by 61% of control levels over a four week period. When considering all of the rats fed the reduced energy diet in the present study, regardless of treatment or Zn levels, a 30% reduction in energy intake resulted in a 40% reduction in body weight gain in comparison to all control animals.

It was previously found that GHi given to rats fed a restricted diet (60% energy) resulted in increased cumulative weight gain when compared to their NSS controls, while IGF-I and IGFBP-3 levels were elevated [20]. Our results confirm that GHi enhanced body weight gain by >18% when rats were fed suboptimally (reduction by 30% in dietary energy). Body weight differences were mainly associated to energy restriction. No body weight differences were evident in Zn restricted rats as long as the energy intake was appropriate.

Increments in IGF-I and IGFBP-3 serum concentrations were clearly linked to the effects of GHi in energy restricted rats. However, our data demonstrated no differences in serum IGF-I and IGFBP-3 concentrations with mild isolated Zn restriction. Thus, reduced Zn intake influenced the action of GHi in IGF-I and IGFBP-3 serum concentrations only when the energy intake was simultaneously restricted, suggesting no role for the suboptimal intake of Zn. It is possible that reducing Zn to only 70% of requirements, with adequate energy, is not enough to cause any significant effects on the hormonal parameters studied. It may be necessary to further reduce dietary Zn before an effect on IGF-I or IGFBP-3 is observed. For example, Zn deficiency in rats fed adequate energy grew poorly and showed reduced serum IGF-I, GH receptor numbers and GH binding proteins [26]. Furthermore, gene expression for both IGF-I and GH receptors in rats were reduced when fed a Zn deficient, adequate energy diet [24].

Altogether, recombinant human growth hormone therapy reduces carbohydrate utilization shifting all metabolic loads to lipid metabolism [30,31]. Moreover, physical activity, normal growth, dietary intake [31] and GH itself have an essential role in body composition [32-35]. For instance, the administration of GHi, promoting lipid utilization, reduces total body fat. In our study our partially energy restricted animals showed a slight increase in body weight gain with GHi treatment. Moreover, we found a slight reduction in the respiratory quotient with GHi administration in those rats on energy restricted diets. This suggests that GHi treatment shifts the metabolic load to lipogenesis, as has been found in a previous study [31,37]. Furthermore, GHi action was not affected by restricted Zn intake as long as the energy intake was not restricted.

The amount of GH administered may have variable effects on body composition in rats. For example, investigators in two previous studies found minimal changes in body fat and fat-free mass in rats fed ad-libitum and administered 0.05 or 0.10 mg/100 g body weight of GHi daily [20,21]. This is suggestive that under ad-libitum or mild energy restriction GHi treatment at or below 0.10 mg/100 g body weight will not change the amount of fat-free mass relative to body fat. It is possible that a higher dosage of GHi will elicit these effects. For example, body fat decreased significantly in similarly fed rats when administered 0.35 mg/100 g body weight of GHi daily [37]. Moreover, the whole body carcass analysis methodology may not be sensitive enough to detect subtle changes in body fat in rats at the lower dosages of GHi used in this and in similar studies [20,21]. This is further evidenced by the lack of changes in energy expenditure and physical activity index observed in rats fed either ad-libitum or energy restricted diets and treated with 0.1 mg/100 g body weight of GHi.

In this study energy restriction resulted in a reduction of 24-hour energy expenditure and physical activity index in rats. Furthermore, these changes were not affected by GHi treatment. This is suggestive that metabolic adaptations occurred due to suboptimal energy intake. Others have found similar metabolic and biochemical changes associated with suboptimal energy intake. For example, erythrocyte NaK-ATPase was reduced in children diagnosed with nutritional dwarfing, a form of suboptimal nutrition that exists for a prolonged period of time [8]. Similar reductions in erythrocyte NaK-ATPase were also found in rats fed energy restricted diets [5]. Moreover, metabolic rate and physical activity were reduced when rats were fed energy restricted diets [43]. The results of all these studies [5,8,20,21,37] suggests that metabolic adaptations occur beginning with mild energy restriction.

Growth deceleration, subsequent growth failure and short stature are the most remarkable consequences of persistent suboptimal nutrition [1-5]. Although adequate energy intake has a preponderant role affecting growth, many other micronutrients play a meaningful role in growth regulation and growth hormone action. Furthermore, extensive animal and human research have already established the association between energy restriction, severe Zn deficiency and growth failure. Therefore, elevated growth hormone levels, along with reduced IGF-I, IGFBP-3 and insulin levels, have been described in severe energy and Zn restrictions [10-18,22-26].

Malnourished humans have increased, whereas rats have decreased serum levels of growth hormone [38]. Decreased growth-hormone levels in starved rats may be caused by increased somatotropin-releasing inhibitory hormone or by reduced stimulation of hypothalamic somatotroph cells by growth-hormone-releasing hormone [39]. Furthermore, serum leptin has been found to play a role in the decline of growth hormone in rats [40]. However, serum insulin growth factor-1 (IGF-I) production in starved rats remains sensitive to growth hormone levels [41]. In previous studies we have validated the rat model for testing various treatments during suboptimal nutrition [5,20,21].

Growth retardation is the most predominant finding in many of the conventional Zn deficiency studies [24-29]. However, limited information exists concerning the effects of suboptimal Zn intake [42] and no one has investigated the effects of GHi administration during experimental suboptimal Zn restriction. Thus, our study showed GHi therapy promoting body weight gain despite suboptimal Zn intake. Consequently, in contrast to most of these studies involving severe Zn deficiency, our data suggest a secondary role of Zn when Zn intake was only restricted to 70% of daily requirements while maintaining adequate energy intake. In addition to weight gain, other GHi metabolic effects were also obtained, such as increases in both IGF-I and IGFBP-3 levels, during Zn restriction if associated with appropriate energy intake.

Our study results give us an idea of the role of Zn in growth during mild malnutrition, which is not clearly understood. The assessment of Zn status is hampered by the lack of a single sensitive and specific biochemical factor [43]. Currently, the clinical method to assess Zn status in children is to measure increase growth velocity in response to Zn supplementation. Controversy exists between studies, where positive effects of Zn supplementation have been found against no effects of supplementation. No effects were found in adolescents with ND and in sub Saharan infants and young children [5,6,12-15] while malnourished infants and children showed increased growth [6-8]. Researchers have suggested that these differences may be attributed to the severity of Zn deficiency, to the dose, frequency of dosing and age of the infants. It has also been observed that in non-stunted infants, Zn supplementation results in increased growth compared with the control group, but with a less pronounced effect. Therefore, researchers have concluded that beneficial effects of Zn supplementation on growth are related to the degree of stunting and of Zn deficiency. Furthermore, it was suggested that Zn supplementation halted the stunting process, due to improved appetite and decreased episodes of gastrointestinal and respiratory disease, in stunted infants in rural Ethiopia [44].

The amount and method of administration of growth hormone, along with the type of feeding regime might cause differences in anabolic responses. For example, healthy normally fed rats injected with 20 micrograms daily of recombinant human growth hormone at the sight of an induced fracture for 10 days showed reduced healing times without changes in bodyweight [45]. In another study, 15 day slow release recombinant human growth hormone laminar implants elicited the same anabolic effects in rats as the daily injectable form but without the associated problems such as renal toxicity and stress at the injection site [46]. Furthermore, a single injection of microencapsulated slow release recombinant human growth hormone in immunosuppressed Hpx rats showed greater long term pharmacological effects such as increased growth rate and IGF-I levels in comparison with daily injections for 35 days [47]. Moreover, there were no differences in the anabolic effects of either rodent or human growth hormone administration in rats [48]. In our study we utilized GHi was injected daily just under the skin (SC) at the back of the neck. The half-life of this type of growth hormone is 124 minutes when utilized in rodents [49]. We have conducted three prior studies of suboptimal nutrition in our laboratory [5,20,21] utilizing the same type of growth hormone. We always observed increased growth and body weight gain without any apparent health problems. It is possible that the short half life of this product prevented any toxic effects in our rats.

## Conclusion

Our results demonstrate that beneficial effects of GHi are obtained during mild energy restriction. Moreover, mild Zn restriction does not have negative effects on body weight gain. Growth hormone therapy does promote body weight gain despite mild energy and Zn restrictions without any apparent effects on metabolic rate and physical activity. Finally, no additive effects between Zn and energy are observed during mild combined restriction of energy and Zn intakes.

### Materials and methods

**Figure 1:**
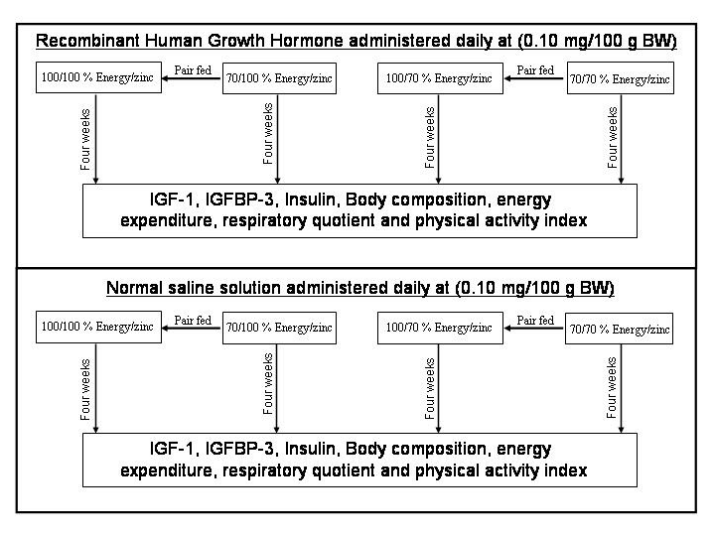
Experimental design and assays

Forty pre-pubertal two week old male Sprague-Dawley rats were studied over a four-week period at the Miami Children's Hospital Research Institute in Miami Florida. The animals were individually housed in wire-bottom stainless steel cages avoiding coprophagia. The experimental design is depicted in Figure 1. Rats were maintained on a 12 hour light/dark cycle beginning at 7:00 AM. Four groups of 10 Sprague-Dawley male rats were fed four different balanced purified 1:1 carbohydrate to fat diets (Purina Mills Test Diets, Richmond, IN) adjusted for energy and Zn according to the following [50]:

1) Diet A – 100% of all nutrients, fed ad-libitum

2) Diet B – 70% energy and 100% of all the nutrients, pair-fed with rats in group A

3) Diet C – 100% energy and all the nutrients but 70% Zn, fed ad-libitum

4) Diet D – 70% of both energy and Zn, and 100% of the rest of the nutrients, pair-fed with rats in group C

Purified diets were used in order to precisely control the amount of nutrients and to eliminate the variability often associated with commercial rat chows. The composition of the experimental diets is shown in Table 1. The control diet (Diet A) was formulated to contain all nutrients, including vitamins and minerals, necessary for normal growth in rats as defined by the National Research Council [50]. The dietary guidelines for rodents were based on rats consuming approximately 60 calories (15 g) of commercial rat chow per day. The formulation of each diet and the related modifications to the energy and Zn levels for the restricted diets (Diets B and D) were based on consumption of similar diets in three previous studies of suboptimal nutrition [5,20,21].

**Table 1:** Composition of experimental diets

Energy/Zinc (%)^1^	100/100	70/100	100/70	70/70
Casein (92% purity)	24.8	24.8	24.8	24.8
DL-Methionine	0.22	0.34	0.22	0.34
Sucrose	15.00	15.00	15.00	15.00
Dextrin	22.70	22.70	22.70	22.70
Lard	8.37	8.37	8.37	8.37
Corn Oil	8.37	8.37	8.37	8.37
Choline bitartrate	0.15	0.22	0.15	0.22
Vitamin mix	0.74	1.12	0.74	1.12
Mineral mix	2.60	3.79	2.60	3.79
Alpha cellulose	17.09	15.33	17.09	15.33
Zinc Carbonate (52% purity)	0.0023	0.0023	0.00162	0.00162

^1 ^= The amount of each ingredient is the percentage of the total diet

The amount of both restricted diets (Diets B and D) fed were based on the amount of control diets (Diets A and C) consumed by pair-fed control rats. For example, the amount of the restricted diet (Diet B) fed was based on the consumption of the rats pair-fed the control diet (Diet A) from the previous day. All nutrients, except energy, were concentrated to compensate for the 30% reduction in food intake. For the rats fed the diet containing adequate energy but with reduced levels of dietary Zn (Diet C), the Zn level was reduced to 8.1 mg/kg of diet, based on the Zn requirements for adult rats (12 mg Zn/kg diet) as set by the National Research Council [50,30]. Both adequate Zn diets (Diets A and B) were formulated to contain 12 mg Zn/kg of diet. The amount of Diet D fed, were both energy and Zn were formulated at 70% of requirements, was based on the previous days intake of rats pair-fed adequate energy but with 70% of Zn requirements (Diet C).

One half of each group were administered a daily dose of 0.1 mg of GHi per 100 grams of body weight [20] (Somatropin rDNA Origin, Humatrope, Eli Lilly & Co. Indianapolis, IN) subcutaneously before 10:00 AM in the morning, while the controls received a similar amount of normal saline solution (NSS), the diluent for GHi.

The percentages of metabolisable energy from fat, carbohydrate and protein were adapted from McCarger et al [51] and approximated those of typical American diets. The proximate analysis and macro-nutrient distribution is shown in Table 2. Because the rats were provided excess protein over the minimal requirement (15% for growth, maintenance and breeding) in the ad-libitum diet (Table 2), it was not necessary to adjust crude protein content for formulation of the restricted diets. All control and restricted rats were consuming approximately 23 and 16% protein, respectively. However, micronutrient levels were maintained at adequate levels by increasing their concentration in the restrictive diets. The percentage of alpha cellulose was reduced to accommodate the increased concentrations of micronutrient mixes (Table 1).

**Table 2:** Proximate analysis of experimental diets

Energy/Zn(%)	100/100	70/100	100/70	70/70
	**Percentage macronutrient composition**			
Protein	22.78	15.95	22.78	15.95
Fat	38.38	38.38	38.38	38.38
Carbohydrate	37.70	26.39	37.70	26.39
	**Percentage contribution to energy**			
Protein	23.21	23.21	23.21	23.21
Fat	38.38	38.38	38.38	38.38
Carbohydrate	38.41	38.41	38.41	38.41
	**Percentage contribution to total intake**			
Energy	100	70	100	70
Zinc	100	100	70	70

Rats consumed deionized water ad-libitum. Furthermore, this water was used for food preparation. The deionized water was found to be zinc free by the Miami-Dade Department of Public Health utilizing graphic furnace atomic absorption spectroscopy. The lower limit of detection was 0.05 ug/l (Trace Analytical Laboratories, Muskegon MI). Weight and estimate of food intake were obtained for each rat daily in the morning prior to GHi and NSS injections. Food fed to the restricted rats was calculated based on the amount of food consumed by the respective pair mates in the ad-libitum fed groups. The amount of food given to the rats paired at 70% of energy restriction was calculated as follows: [(food consumed by the ad-libitum rat during the previous day/weight of this rat in the previous day) × (0.7) × (Current weight of the rat for which the food is estimated)]. Weight and food intake were recorded daily. After four weeks of the experimental period all rats were anesthetized with Nembutal (30–50 g/kg BW) and sacrificed by cardiac puncture in the morning at the same time of recording of body weight and GHi and NSS injections. Furthermore, all rats were fasted that same morning to prevent any effects of absorbed nutrients on hormonal assays. The last injection of GHi and NSS occurred the prior morning thus eliminating any effect of these injections on the results obtained from blood samples. Blood samples for serum IGF-I, IGFBP-3 and Insulin were drawn and carcasses were frozen at -20 degrees C for future measurements of body composition using Folch's analysis method for fat extraction [52]. Samples were not frozen more than one week prior to analysis.

All experimental protocols were reviewed and approved by the Miami Children's Hospital Institutional Animal Care and Use Committee.

### Hormonal assays

Blood samples were spun and corresponding serum samples fractionated in aliquots. All serum samples were frozen at -20 degrees C for no more than one week for subsequent analysis. Each hormonal assay was run simultaneously on all samples.

Serum IGF-I concentration was measured by double antibody radioimmunoassay (RIA). Samples were prepared by acid-ethanol extraction followed by cryoprecipitation using a kit from Nichols Institute Diagnostics (San Juan Capistrano, CA). This procedure minimized the interference of IGF-I binding proteins at the time of the assay. The standard curve was linear between 5 and 600 ng/ml with a Coefficient of Variation of 3%. The sensitivity of this radioimmunoassay was equal to 0.06 ng/ml. The intra-assay coefficient of variation was equal to 2.4 and 3% at concentrations of 0.5 and 0.9 ng/ml, respectively. The inter-assay variance at 0.5 ng/ml and 0.8 ng/ml were equal to 5.2 and 8.4%, respectively.

Serum IGFBP-3 level was measured by double antibody RIA using a kit from Diagnostic Systems Laboratories, Inc. (Webster, TX). The standard curve was linear between 2.5 and 100 ng/ml with a Coefficient of Variation of 3.2%. The minimum detection limit was 0.5 ng/ml. The intra-assay precision at 82.7, 27.5 and 7.3 ng/ml was 1.8, 3.2 and 4%, respectively. The inter-assay precision at 80, 21, and 8 ng/ml was 1.9, 0.5 and 0.6%, respectively.

Serum Insulin level was measured by double antibody RIA using a kit from ICN Pharmaceuticals, Inc. (Costa Mesa, CA). The standard curve was linear between 5.5 and 310 mIU/ml with a Coefficient of Variation of 8%. The sensitivity was 1.5 mIU/ml. The intra-assay variation were determined for the following concentrations at 18.2, 36.5 and 91.5 mIU/ml and were 8.2, 4.2 and 5.4%, respectively.

### Metabolic assessment

**Figure 2:**
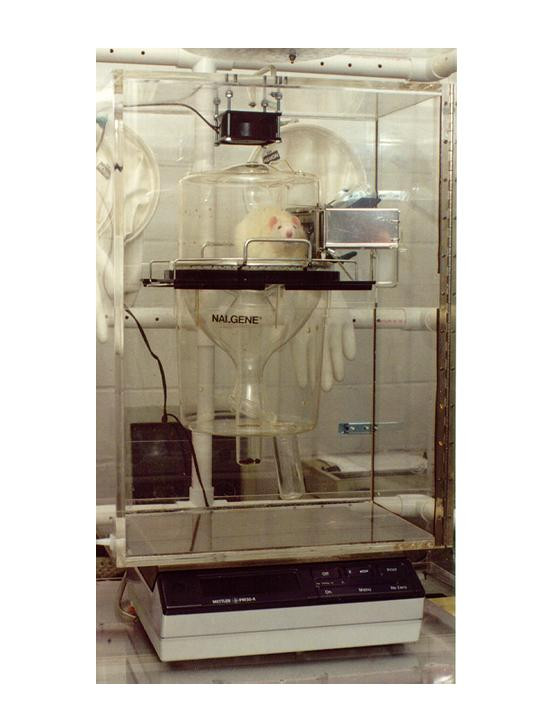
Rodent EMTAC enclosure that was used to obtain measurements of 24-hour energy expenditure and the index of physical activity. Note the placement of the enclosure on the balance for measurement of the index of physical activity.

Each rat was weighed and had 24-hour energy expenditure (24-h EE; kcal/100 g BW), respiratory quotient (RQ;VCO_2_/VO_2_) and an index of physical activity (PA; oscillations/min/kg BW) measured in the Enhanced Metabolic Testing Activity Chamber (EMTAC). The main analytical unit of this instrument was developed to be suitable for various applications in both humans and animals for comprehensive measurements of energy expenditure and physical activity [53,54]. For this study, the EMTAC was retrofitted with a 72 liter plexiglass rodent enclosure (Figure 2). This maintained the rodent's environment thus eliminating the need to acclimate the rat to a different metabolic chamber. Measurements of 24-h energy expenditure and the index of physical activity consisted of first placing the rat, along with the appropriate experimental diet and deionized water, in a Nalgene metabolic cage. This cage was inserted into the EMTAC rodent enclosure at 10:00 AM and energy expenditure determinations were done by measurement of oxygen and carbon dioxide exchange within the enclosure. The same light/dark cycle used in the rodent facility was maintained. The physical activity index was obtained by placing the entire rodent enclosure on a balance that was connected to the EMTAC unit. The oscillations in weight (g), generated by movements of the rat, were read from the balance and utilized to calculate an index of physical activity expressed as oscillations in weight (g)/minute/kg body weight of the rat. For these calculations the software calculated the body weight of the rat in kilograms. The formulas used to calculate energy expenditure and physical activity index using the EMTAC have been described previously [53,54,34]. Energy expenditure was expressed per 100 g body weight to factor out the effects of body weight changes on metabolic rate.

### Body composition analysis

Body composition analysis included total body water content (TBW), fat-free mass (FFM) and body fat (BF) determinations. This procedure comprised three different steps: 1) carcass homogenization, 2) water content and dry weight determinations and 3) fat content determination by fat extraction.

The first step consisted of autoclaving each animal carcass for one hour at 15.3 psi and 120 degrees C in order to facilitate carcass homogenization. Each carcass was individually placed in a large beaker with covered tops with a known amount of distilled water. Each carcass was allowed to cool off overnight and then homogenized using a PowerGen700 blender. Triplicate aliquots of the homogenate were frozen at -20 degrees C for subsequent analysis. The chemical analysis of each homogenized carcass was carried out in triplicate and mean values of these triplicate samples were taken as ultimate values of TBW and BF content. The second step consisted of drying samples overnight at -380 mm Hg at 40 degrees C in a vacuum oven to determine water content. The difference between petri dish weight before and after overnight water extraction was considered as the dry weight of the carcass sample.

The third step consisted of determining BF content by a modified Folch's method [52] for fat extraction. All samples were analyzed following this technique's protocol which comprised two different procedures. In the first procedure, lipids were extracted from the homogenate by adding a 2:1 methanol-chloroform mixture. Each sample was separately filtered and a 5-fold volume of distilled water was added to separate lipids from non-lipid substances. This mixture was centrifuged for 15 minutes at 3000 rpm at three degrees C, producing three separated layers. The clear upper layer contained a mixture of methanol and water. The fluffy middle layer contained non-lipid substances and the clear lower layer contained a mixture of tissue lipids and chloroform. This bottom layer containing lipids and chloroform was isolated by removing the upper and the middle layers by vacuum aspiration. During the second procedure, all samples containing only the remaining bottom layer were dried overnight at -380 mm Hg at 40 degrees C in a vacuum oven, thus allowing chloroform evaporation and subsequent lipid separation. The difference between the tubes weight before and after fat extraction was taken as grams of fat content [55].

Fat-free mass was calculated by subtracting BF percentage from total body mass and expressed as percentage of total body mass.

### Statistical analysis

The effects of GHi on body weight gain, FFM, BF, TBW, Insulin, IGF-I and IGPBP-3 within each dietary treatment group (GHi vs. NSS) were determined by Independent t-test. A similar analysis was utilized to compare changes in 24-hour energy expenditure and physical activity between all ad-libitum and energy restricted (70%) rats. The interaction between diet and hormone treatments across all diet groups was determined by 2-way ANOVA with least significant difference (LSD). Simple size was based on the results of three prior studies of suboptimal nutrition in rats [5,20,21]. In these studies five rats per individual treatment was enough to detect significant differences in body weight gain, the major parameter studied, at p < 0.05. The rest of the parameters were measured in order to explain the changes of body weight gain for each treatment group. All data are presented as mean ± standard deviation (SD) unless otherwise noted.

## Abbreviations

ANOVA = Analysis of Variance

BF = Body Fat

FFM = Fat-free mass

GH = Growth hormone

Hg = Mercury

IGF-I = Insulin-like Growth Factor-1

IGFBP-3 = Insulin-like Growth Factor Binding Protein-3

NSS = Normal Saline Solution

ND = Nutritional dwarfing

PSI = Pounds per square inch

rhGH = recombinant human growth hormone

GHi = exogenous immunoreactive growth hormone

Na^+^K^+^-ATPase = Sodium-potassium ATP transporter enzyme

TBW = Total body water

Zn = Zinc

BW = Body weight

SD = Standard deviation

24-h EE = Twenty-four hour energy expenditure

RQ = Respiratory quotient

## Competing interests

The author(s) declare that they have no competing interests.

## Authors' contributions

Dr. Russell Rising has contributed to the design of the experiment and conducted the data analysis. Furthermore, he either participated in some of the actual data acquisition or supervised pediatric research fellows in this regard. He also assisted in the preparation of the small grants necessary for funding of this project. Finally, he also assisted in the writing and editing of this manuscript.

Dr. Fima Lifshitz contributed to the preparation of the manuscript and assisted with data analysis. He also edited some of the grant proposals necessary for the financial support of this study. Both authors were involved in the final editing of this manuscript.
